# Effects of physical activity trajectories on transitions across cognitive states: A multinational cohorts study

**DOI:** 10.1093/geroni/igaf122.2033

**Published:** 2025-12-31

**Authors:** Chenlu Hong, Yanan Luo

**Affiliations:** Peking University, Beijing, Beijing, China (People’s Republic); Peking University School of Public Health, Beijing, Beijing, China (People’s Republic)

## Abstract

Abstract Background Identifying modifiable factors that prevent dementia progression is crucial. However, the impact of long-term physical activity (PA) trajectories on cognitive transitions remains understudied. This study examines the association between PA trajectories and cognitive state transitions in late life. Methods We analyzed data from HRS, ELSA, SHARE, MHAS (2004–2018), and CHARLS (2011–2018). PA trajectories were classified into two groups using GBTM based on PA intensity and frequency. A three-state model was applied (State 1 = Not Cognitively Impaired; State 2 = Mild Cognitive Impairment (MCI); State 3 = Dementia). The MSM model assessed cognitive transitions, with PA trajectory as a covariate to measure the HRs. A meta-analysis provided an overall effect size across cohorts. Results A higher PA trajectory was associated with a 25% lower risk of transitioning from no impairment to MCI (pooled HR = 0.75; 95% CI: 0.65–0.87) and a reduced risk of MCI progressing to dementia (pooled HR = 0.87; 95% CI: 0.77–0.98), but had no significant effect on cognitive recovery from MCI. Individual study results were largely consistent, though some heterogeneity existed. HRS, SHARE, and MHAS showed significantly lower risk of State 1 to State 2 transition. Only HRS showed a reduced risk of State 2 to State 3, while ELSA reported a significantly higher likelihood of cognitive recovery. Conclusion Our multinational study confirms the protective effect of higher PA trajectories in delaying MCI and dementia onset. Findings suggest even physically inactive individuals may achieve neurocognitive benefits by increasing PA after age 60.

